# Stage I seminoma: treatment outcome at King Hussein Cancer Center in Jordan

**DOI:** 10.1186/1471-2490-12-10

**Published:** 2012-04-24

**Authors:** Jamal Khader, Ahmed Salem, Yazan Abuodeh, Abdelateif Almousa, Naim Farah, Fadwa Abdelrahman

**Affiliations:** 1 Department of Radiation Oncology, King Hussein Cancer Center, Queen Rania Alabdulla Street, Amman 11941, Jordan; 2 Department of Surgical Oncology- Division of Urology, King Hussein Cancer Center, Queen Rania Alabdulla Street, Amman 11941, Jordan

## Abstract

**Background:**

The aim of this report is to address treatment outcomes of patients with early-stage seminoma in a single institution with special reference to patients with history of surgical violation of the scrotum.

**Methods:**

Seventy four patients with pure seminoma were treated at King Hussein Cancer Center (Amman, Jordan) between 2003 and 2010. All patients underwent orchiectomy. All but 3 patients received adjuvant radiotherapy. Patients who underwent surgical violation of the scrotum prior to referral were managed by further excision or irradiation of the scrotal scar. The follow-up ranged from 1 to 200 months (mean, 33 months).

**Results:**

At the time of follow-up; all but one patient remain alive. The 3-year relapse-free survival for the entire cohort was 95.9%. Three patients developed relapse, all of whom received adjuvant irradiation following inguinal orchiectomy and initially harbored tumors larger than 4 cm upon pathological examination. Median time to relapse was 14 months (range, 8–25 months). None were associated with elevated tumor markers prior to detection of relapse. All but one patient were successfully salvaged by chemotherapy.

**Conclusions:**

Our results confirm the excellent prognosis of patients with early-stage seminoma treated by orchiectomy and adjuvant radiotherapy in a developing country. Although all patients who developed relapse demonstrated adverse pathological findings upon initial assessment, no consistent predictor of relapse was found. Scrotal scar re-excision or irradiation in patients with prior history of surgical violation of the scrotum are effective measures in preventing local failure.

## Background

Testicular cancer is the most common malignancy in men 20 to 40 years of age [1]. More than half of patients with testicular cancer are found to harbor a seminoma [2]. Over the past years, there has been a continuously increasing incidence of testicular seminoma in the Western world and Japan [3,4]. In the United States, 8480 new cases and 350 deaths were expected in 2010 [5]. Seventy to eighty percent of seminoma patients present with stage I disease [2]. High inguinal orchiectomy is the standard initial treatment [1]. Due to the lack of comparative randomized trials, the choice of the most appropriate adjuvant management approach remains controversial [2]. Adjuvant radiotherapy is associated with a low rate of relapse set at 3-4% and remains the standard treatment in the United States and the most frequently used adjuvant modality in Europe [2]. Regardless of management strategy, virtually all patients are cured [5].

Disappointingly, there is paucity of data assessing achievable outcomes of seminoma patients outside developed countries. The aim of this report is to address treatment outcomes in patients with early-stage seminoma in a developing country with special reference to patients who underwent surgical violation of the scrotum.

## Methods

Between January 2003 and December 2010; seventy four patients with histologically-confirmed pure seminoma (classical and anaplastic subtypes) were treated at King Hussein Cancer Center (KHCC) (Amman, Jordan). Records were electronically retrieved and retrospectively reviewed following acquisition of KHCC Institutional Review Board approval (approval number; 10 KHCC 55). Research conducted in this study was in compliance with the Helsinki Declaration. Exhaustive chart analysis was performed in an attempt to extract data pertaining to pathological characters, clinical stage, treatment, disease outcome and survival. Written informed consent was obtained from all patients. Patients were evaluated with thorough history, complete physical examination and computed tomography (CT) of the chest, abdomen and pelvis. Pathological diagnosis was confirmed by examining/re-examining surgical specimens obtained via ochiectomy by a staff pathologist at KHCC. Patients were classified according to the 2002 American Joint Committee on Cancer-International Union Against Cancer classification [6] and were included for analysis if they exhibited stage I disease with no clinical and/or radiological evidence of distant metastases at the time of diagnosis. Cases were discussed at a joint care conference prior to therapeutic decisions and delivery of care. Initial therapy consisted of inguinal orchiectomy followed by adjuvant radiotherapy in most patients. Complete blood counts and appropriate serum markers (β-human chorionic gonadotropin (β-HCG), lactate dehydrogenase (LDH), and α-feto protein (AFP)) were obtained in a proportion of patients prior and subsequent to surgery and at the time of follow-up. Long-term fertility data were not available for this report; however, all patients were offered sperm banking prior to initiation of radiotherapy.

**Figure 1:**
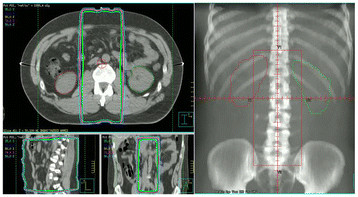
Isodose distributions and antro-posterior digitally reconstructed radiograph of a typical PA field.

Radiation therapy was delivered via an appropriate energy Elekta linear accelerator (Elekta Oncology Systems, Crawley, UK). Conventional or CT simulation was performed in all patients. The para-aortic and dog-leg fields were treated via a two-field parallel-opposed technique to a total dose ranging from 2000 to 2500 cGy given over a period of 2 to 3 weeks (daily fractions in 5 consecutive days of the week). Standard, previously published, field designs were utilized [7]. Figure 1 shows a typical para-aortic field.

Follow-up visits were conducted every 3 months for the first 2 years after radiotherapy and every 6 months thereafter. Clinical examination and analysis of AFP, LDH and β-HCG levels were recorded if available. CT scans of chest, abdomen and pelvis were performed twice a year for the first 2 years and annually thereafter for a total of 10 years. Acute and late radiotherapy-related side effects were recorded during radiotherapy and at each follow-up visit, respectively. Follow-up was in the form of a patient visit to a staff clinician at KHCC or retrieval of institutional-tumor registry and national data archives.

## Results

The median age of our study population was 34 years (range, 17–51 years). All patients underwent orchiectomy; 67 (90.5%) of which were performed via the inguinal approach. Seven patients were referred to our center following scrotal-approach orchiectomy. These patients were treated by further excision of the surgical scar (3 patients) or local-field irradiation of the scrotal scar delivered via an additional electron beam (4 patients). Fifty nine (79.7%) patients harbored T1 while 15 (20.3%) harbored T2 disease. None of the included patients had documented previous history of ipsilateral pelvic surgery. All but 3 patients received adjuvant radiotherapy via para-aortic fields in 63 (88.7%) patients or dog-leg fields in 7 patients (9.9%) (Table 1). The mean time form orchiectomy to initiation of radiotherapy was 57.9 days. Acute radiotherapy-related side effects were mild in all patients, while none demonstrated late toxicity at the time of follow-up. The length of follow-up -for the whole cohort- ranged from 1 to 200 months, with a mean follow-up period of 33 months. The median time of follow-up for the 3 patients who were kept on active surveillance was 37 months (range, 12–77 months). None of whom had developed disease relapse.

**Table 1:** Patient characteristics of the entire cohort

**Patient characteristic**	**Number (%)**
Type of orchiectomy	
- Inguinal approach	67 (90.5%)
- Scrotal approach	7 (9.5%)
Re-excision of scrotal scar	3
Local radiation to scrotal scar	4
Laterality	
- Right	43 (58.1%)
- Left	29 (39.2%)
- Bilateral	1 (1.35%)
- Unknown	1 (1.35%)
Histology	
- Classical seminoma	68 (91.9%)
- Anaplastic seminoma	6 (8.1%)
Tumor stage (assessable in all patients)	
- T1	59 (79.7%)
- T2	15 (20.3%)
Adjuvant radiotherapy 1	71 (95.9%)
- Para-aortic field	63 (88.7%)
20 Gy/10Fx	48
25 Gy/15Fx	15
- Dogleg field	7 (9.9%)
20 Gy/10Fx	4
25 Gy/15Fx	3
Relapse	3 (4%)
- Local	0 (0%)
- Regional	1 (1.3%)
- Distant	2 (2.7%)

1: One patient received radiation at outside facility, no further details were available for this patient.

**Figure 2:**
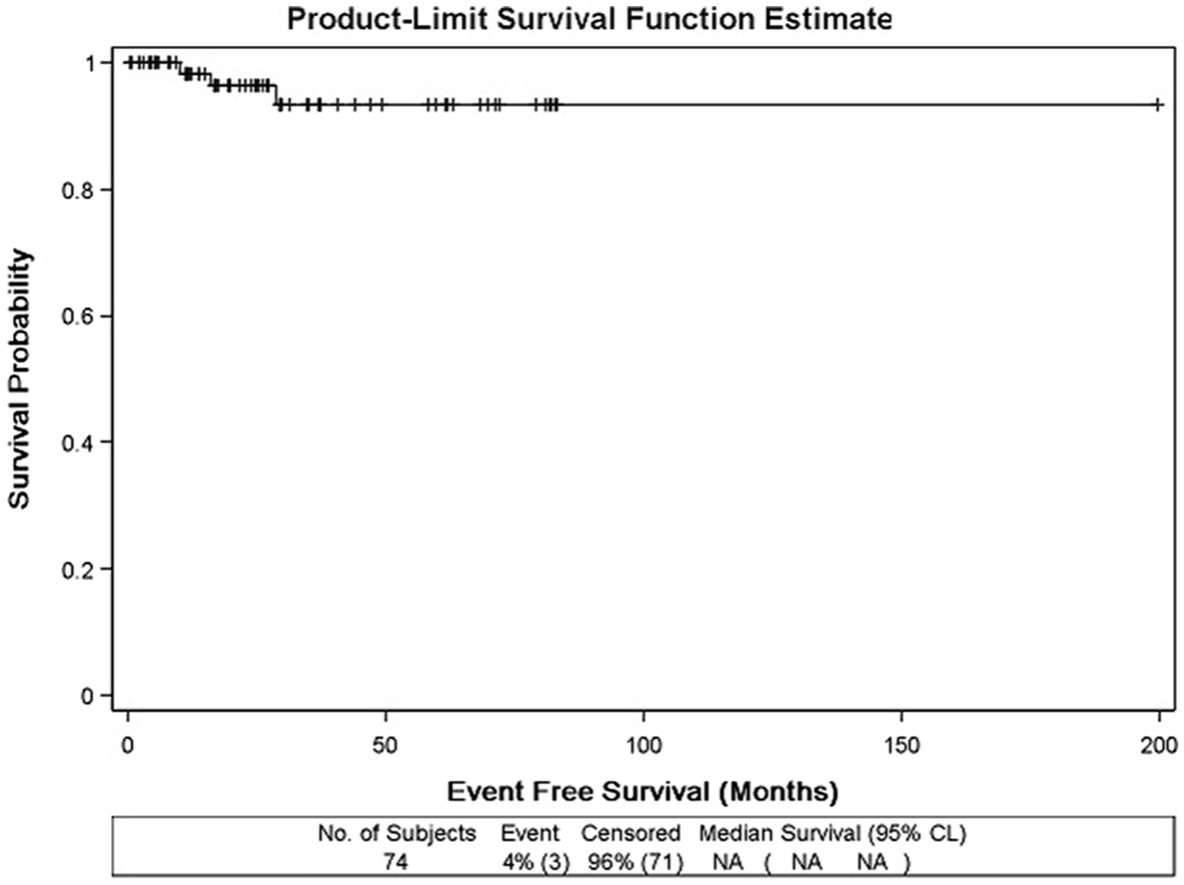
Progression free survival of the entire cohort.

**Figure 3:**
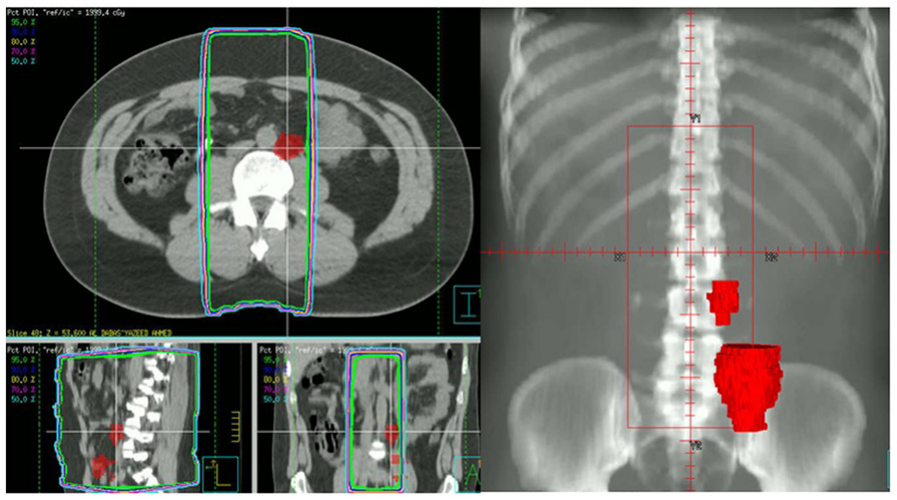
Co-registration of CT scan demonstrating recurrence with the panning CT scan; recurrence is in-field.

At the time of follow-up; all but one patient were alive. The 3-year relapse-free survival for the entire cohort was 95.9% (Figure 2). Three patients developed relapse, all of whom received adjuvant irradiation following inguinal orchiectomy and initially harbored tumors larger than 4 cm upon pathological examination. Median time to relapse was 14 months (range, 8–25 months). The site of failure was regional in 1 patient and distant in 2 patients. CT scan of the abdomen in the patient harboring regional recurrence was compared with simulation CT demonstrating that this recurrence was actually in-field (Figure 3). Relapse was initially detected via clinical examination in 1 and computed tomography in 2 patients. None were associated with elevated tumor markers prior to detection of relapse (Table 2). Preoperatively, eleven patients (13.1%) demonstrated abnormal β-HCG while 2 patients (2.4%) had abnormal LDH. However, none of the patients with previous history of abnormal tumor markers developed relapse. All but one patient were successfully salvaged by chemotherapy (details of the administered chemotherapy protocols are mentioned in Table 2). After a median follow-up of 28 months (range, 7–74 months), and subsequent to re-excision or local irradiation of the scrotal scar, none of the 7 patients with prior history of surgical violation of the scrotum developed relapse (Table 3).

**Table 2:** Characters of the 3 patients who developed relapse

**No.**	**Age (years)**	**T stage**	**Ochiectomy approach**	**Adjuvant radiotherapy field (dose)**	**Pathological risk features**	**Preoperative tumor markers**	**Postoperative tumor markers**	**Abnormality if tumor marker levels prior to relapse**	**Method of detection of relapse**	**Site of relapse**	**Time from radiotherapy to relapse (months)**	**Treatment**	**Outcome (time at follow-up from relapse)**
1	40	T1	Inguinal	Para-aortic (20 Gy/10Fx)	4.6 cm tumor	Normal	Normal	No	CT scan	Left hilum	25	Chemotherapy (BEPx4 cycles)	ANED (32 months)
2	34	T2	Inguinal	Para-aortic (20/10Fx)	6 cm tumor	Not available	Normal	No	CT scan	Retro- peritoneal LN	8	Chemotherapy (BEPx4 cycles, TIPx4 cycles, VIPx4 cycles, GOPx8 cycles)	DDD (25 months)
3	38	T2	Inguinal	Para-aortic (20/10Fx)	8 cm tumor	Normal	Normal	No	Clinical examination	Supra- clavicular LN	14	Chemotherapy (BEPx4 cycles)	AWD (12 months)

BEP: Bleomycin, Etoposide, Cisplatin, TIP: Paclitaxel, Ifosfamide, Cisplatin, VIP: Etoposide, Ifosfamide, Cisplatin, GOP: Gemcitabine, Oxaliplatin, Paclitaxel, ANED: Alive no evidence of disease, AWD: Alive with disease, DDD: Dead due to disease.

**Table 3:** Details of the 7 patients with prior history of surgical violation of the scrotum and their outcome

**No.**	**Age (years)**	**T stage**	**Surgical approach**	**Histology**	**Pathological risk features**	**Preoperative tumor markers**	**Postoperative tumor markers**	**Adjuvant radiotherapy field (dose)**	**Management of scrotal scar**	**Outcome (time at follow-up from relapse)**
1	27	T1	Scrotal biopsy followed by inguinal orchiectomy	Classical	5 cm tumor	Normal	Normal	Dog-leg (25 Gy/15Fx)	Local irradiation (25 Gy/15Fx)	ANED (74 months)
2	44	T1	Scrotal orchiectomy	Classical	None	Normal	Normal	Para-aortic (20 Gy/10Fx)	Re-excision of scar	ANED (20 months)
3	30	T1	Scrotal biopsy followed by inguinal orchiectomy	Classical	6 cm tumor Rete testis invasion	Normal	Normal	Dog-leg (25 Gy/15Fx)	Local irradiation (25 Gy/15Fx)	ANED (53 months)
4	44	T1	Scrotal biopsy followed by inguinal orchiectomy	Classical	Not available	Not available	Normal	Dog-leg (20 Gy/10Fx)	Re-excision of scar	ANED (28 months)
5	45	T1	Scrotal exploration followed by inguinal orchiectomy	Classical	Not available	Not available	Normal	Dog-leg (25 Gy/15Fx)	Re-excision of scar	ANED (7 months)
6	40	T1	Scrotal orchiectomy	Classical	None	Normal	Normal	Dog-leg (20 Gy/10Fx)	Local irradiation (20 Gy/10Fx)	ANED (12 months)
7	38	T1	Scrotal biopsy followed by inguinal orchiectomy	Classical	8 cm tumor	Not available	Normal	Dog-leg (20 Gy/10Fx)	Local irradiation (20 Gy/10Fx)	ANED (37 months)

ANED: Alive no evidence of disease.

## Discussion

**Figure 4:**
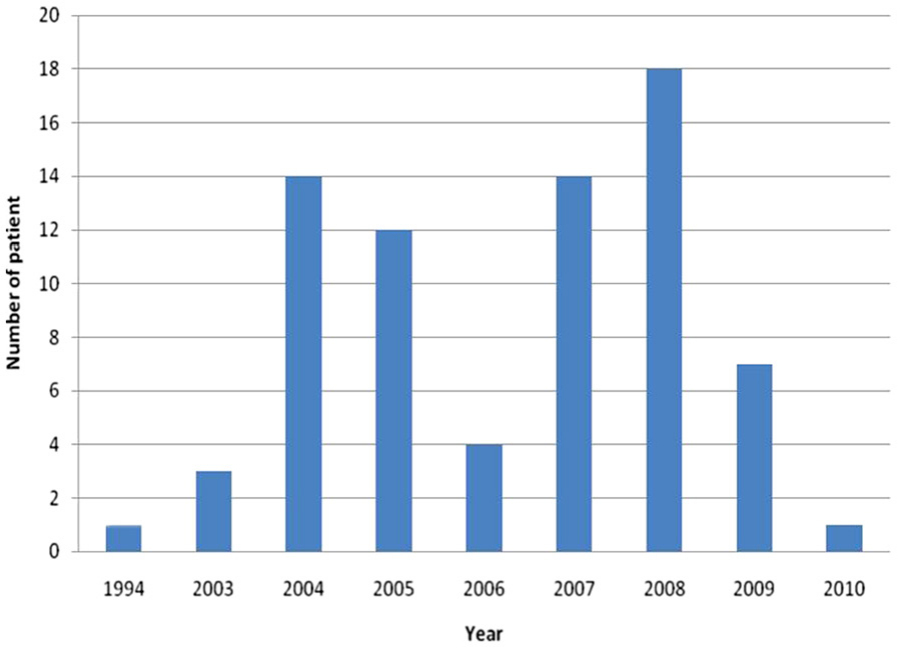
Number of patients diagnosed per year.

Forty four testicular tumor cases were reported in the Jordanian national cancer registry in 2008. Although these neoplasms came short from ranking among the 10 leading cancer subtypes amongst males overall, testicular tumors ranked 7th and 5th amongst males aged 0–19 and 20–49 years, respectively [8]. Since there are no detailed epidemiological studies addressing seminoma in Jordan, we have compiled a chart depicting the temporal distribution of stage I seminoma cases treated at our center over the years of this study (Figure 4).

In this small series; we demonstrated the excellent prognosis for patients with early-stage seminoma treated by orchiectomy and adjuvant radiotherapy or active surveillance in a developing country. Unfortunately, there are no previously published reports assessing outcomes of seminoma patients in Jordan and as such we are unable to compare our results with those of another series. Furthermore, to the best of our knowledge, there is only one published study addressing testicular seminoma in a Middle Eastern country [9]. This report -published in 1986- included a small number of patients treated in a Saudi Arabian center prior to the implementation of modern therapeutic guidelines. Nonetheless, and while acknowledging the limitations of our study including the small sample size and the short follow-up period, we report excellent outcomes in lieu with previously published Western reports.

Radical orchiectomy with ligation of the spermatic cord at the level of the internal inguinal ring is considered the standard treatment of seminoma [2]. Scrotal violation should strongly be avoided, and as such, testicular biopsy is not advised in patients with solid testicular tumors [10,11]. Disappointingly, patients are still occasionally referred to our center with prior history of surgical violation of the scrotum consequent to exploration or biopsy. In our series, these patients were managed by either re-excision or local irradiation to the scrotal scar. After a median follow-up of 28 months (range, 7–74 months), none of these patients developed relapse. This highlights the effectiveness of these approaches in preventing local recurrence.

Para-aortic radiotherapy is considered the standard adjuvant treatment of early stage seminoma with disease-specific survival rates approaching 100% [12,13]. The TE10 trial demonstrated the non-inferiority of para-aortic versus dogleg radiotherapy fields [7]. Most of the patients in our case series were treated by para-aortic fields and only a small proportion (9.9%) received dog-leg fields. In 2005, results of the TE18 trial were published and clearly demonstrated the non-inferiority of lower (20 Gy) as opposed to higher dose (30 Gy) para-aortic radiotherapy [14]. Since then, we have adopted the recommendation of this trial and 52 out of 70 (74.3%) patients with known radiotherapy details received 20 Gy/10Fx.

Although the para-aortic field has emanated as the standard radiation approach in seminoma patients [15], Power et al. [16] reported the occurrence of ipsilateral pelvic relapse in 3 patients that would otherwise have been avoided if the radiation field was extended. In our case series, one out of the 3 patients who developed relapse harbored regional disease. In this patient, para-aortic lymph node metastasis was detected via abdominal CT scan 8 months after the completion of radiotherapy. Co-registration of this image with the radiotherapy simulation CT demonstrated that the relapse was actually in-field. Detailed review of baseline abdominal CT was undertaken in this patient and failed to reveal borderline and/or grossly enlarged or suspicious pelvic or para-aortic masses. The short time interval (from completion of radiotherapy till the appearance of regional relapse) and the spatial location of relapse (inside the radiation filed) support the fact that this patient actually harbored a biologically aggressive tumor from the start.

Due to the overwhelming success of the treatment and the subsequent long-term survival, there has been growing interest in decreasing treatment-related morbidity in seminoma patients [3]. Long-term complications of radiotherapy include infertility and induction of secondary malignancy [1]. Travis and colleagues [17] reported a 1.43 observed-to-expected ratio of developing a second tumor in 29,000 long-term seminoma survivors. Although the absolute number of radiation-induced second malignancy is low [18], this has led several investigators to attempt substituting radiotherapy for chemotherapy in these patients. The TE19 trial demonstrated the non-inferiority of single-injection carboplatin over para-aortic and/or dogleg radiotherapy at a dose of 20 to 30 Gy [19]. However, chemotherapy also comes at the cost of undeniable side-effects. Nonetheless, interest in active surveillance has largely been driven by concerns about secondary malignancies associated with radiotherapy [17]. Subsequent to the short follow-up period and the retrospective nature of this case series, we cannot address the occurrence and/or incidence of second malignancies in our patient population.

Several reports have addressed the safety of surveillance in the management of stage I seminoma [20]. Although relapse rates of 10-30% have been narrated in patients with stage I seminoma [10,21,22], relapses are usually detected early and salvage therapy is usually successful with long-term survival [1]. However, since one of the prerequisites of recommending active surveillance in patients with stage I seminoma is strict patient compliance [23], one could argue against such approach in developing countries such as Jordan where patient compliance is still disappointingly low. In our series, none of the patients under active surveillance developed relapse. However, due to the small number of patients (3 patients), we cannot draw conclusions on the applicability and safety of active surveillance in the management of seminoma patients in our community.

Although no clear predictors of relapse were found, lymphovascular invasion, rete testis invasion and tumor size have all been reported to significantly increase the risk of relapse in patients with seminoma [24]. In our series, all 3 patients presenting with relapse demonstrated large tumors on initial pathological assessment (4.6, 6, 8 cm). Nonetheless, no clear predictors of relapse were found in our patient population.

Investigators have indicated that more than half of patients with relapse initially exhibit indicative symptoms and abnormal findings on physical examination highlighting the importance of patient education and meticulous medical examination [12]. Less than 50% of seminoma relapses present with radiological abnormalities [25]. Furthermore, most cases of relapse are discovered in the first 2 years after treatment. Our study confirms this observation. All the 3 cases of relapse, in our series, were diagnosed 8, 14, and 25 months after completion of radiotherapy. However, late relapse –up to 10 years after treatment- has been previously reported [11]. Nonetheless, no evidence exists supporting the utilization of routine follow-up with computed tomography beyond 3 years of treatment [12].

## Conclusions

Our results confirm the excellent prognosis for patients with early-stage seminoma treated by orchiectomy and adjuvant radiotherapy or active surveillance in a developing country. Although all patients who developed relapse demonstrated adverse pathological findings upon initial assessment, no consistent predictor of relapse was found. We have demonstrated the effectiveness of scrotal scar re-excision or irradiation in patients with prior history of surgical violation of the scrotum consequent to exploration or biopsy.

## Competing interests

The authors declare that they have no competing interests.

## Author’s contributions

JK, AS, FA conceived the study and drafted the manuscript. All authors read and approved the final manuscript.

## Pre-publication history

The pre-publication history for this paper can be accessed here:

http://www.biomedcentral.com/1471-2490/12/10/prepub
